# Research Note: Junctional adhesion molecule A is expressed in epithelial cells of the crypt and villi whereas junctional adhesion molecule 2 is expressed in vascular cells

**DOI:** 10.1016/j.psj.2023.102693

**Published:** 2023-04-05

**Authors:** E.A. Wong, S.R. Kinstler

**Affiliations:** School of Animal Sciences, Virginia Tech, Blacksburg, VA 24061, USA

**Keywords:** JAMA, JAM2, in situ hybridization, tight junction, intestine

## Abstract

A functional intestinal barrier is essential for a healthy intestine. This barrier includes an apical tight junctional complex between adjacent intestinal epithelial cells. The tight junctions (**TJ**) are multiprotein junctional complexes that consist of a number of members of the occludin, claudin, zona occludens, and junctional adhesion molecule families. The mRNA expression of junctional adhesin molecule A (**JAMA**) and junctional adhesion molecule 2 (**JAM2**) are 2 TJ mRNAs that are often used to assess intestinal barrier integrity. The objective of this study was to use in situ hybridization to identify cells that express JAMA and JAM2 mRNA in the small intestine of chickens. In the jejunum of a 21 d old broiler, JAMA mRNA was highly expressed in the epithelial cells of the villi and crypt. By contrast, JAM2 mRNA was located in the vascular system in the center of the villi and in the lamina propria. These results demonstrate that JAMA and not JAM2 is the appropriate gene to use when assessing TJ between intestinal epithelial cells.

## INTRODUCTION

The tight junction (**TJ**) is a multiprotein junctional complex that forms a physiological barrier between epithelial cells, maintains integrity of the cell layer and plays an essential role in the regulation of paracellular transport (Zihni et al., 2016). The TJ contains a series of transmembrane proteins, including occludin (**OCLN**), claudins (**CLDN**), zona occludens (**ZO**) and junctional adhesion molecules (**JAM**) (Zihni et al., 2016; Awad et al., 2017). Two members of the JAM family, JAMA and JAM2, are often used to assess the functionality of the TJ. JAM2 is the most commonly assayed JAM mRNA in chickens (e.g., Barekatain et al., 2019; Goo et al., 2019; Park et al., 2020; Tabler et al., 2020; Bilal et al., 2021; Lin et al., 2022). In mammals, JAMA, which is also known as the F11 receptor, is expressed in intestinal epithelial cells and regulates barrier function. JAM2, which is the same as JAMB, is not expressed in epithelial cells but is restricted to endothelial cells, such as vascular cells (Hartmann et al., 2020). The cellular localization of JAMA and JAM2 mRNA has not been determined in chickens. The objective of this project was to use in situ hybridization **(ISH**) to identify cells expressing JAMA and JAM2 mRNA in the small intestine of chickens in order to determine which is the key gene for assessing intestinal barrier integrity.

## MATERIALS AND METHODS

Ross broilers were raised at the Southern Poultry Research Group (Watkinsville, GA). Chicks were provided ad libitum water and a commercial-type broiler starter diet formulated according to NRC guidelines. Animal care practices conformed to the Guide for the Care and Use of Agricultural Animals in Agricultural Research and Teaching (FASS, 2020). At d 21, jejunal samples (n = 4) were collected, fixed in formaldehyde and embedded in paraffin. Six micron serial sections were cut with a microtome and placed on Superfrost plus glass slides. Serial sections were analyzed by ISH using custom probes to JAMA (Accession number NM_001083366.1) and JAM2 (Accession number NM_001006257.1) and the RNAscope 2.5 HD Assay-RED kit (Advanced Cell Diagnostics, Newark, CA). Fluorescent images were captured using a Nikon Eclipse 80i microscope and Ds-Ri2 digital camera.

## RESULTS AND DISCUSSION

**Figure 1:**
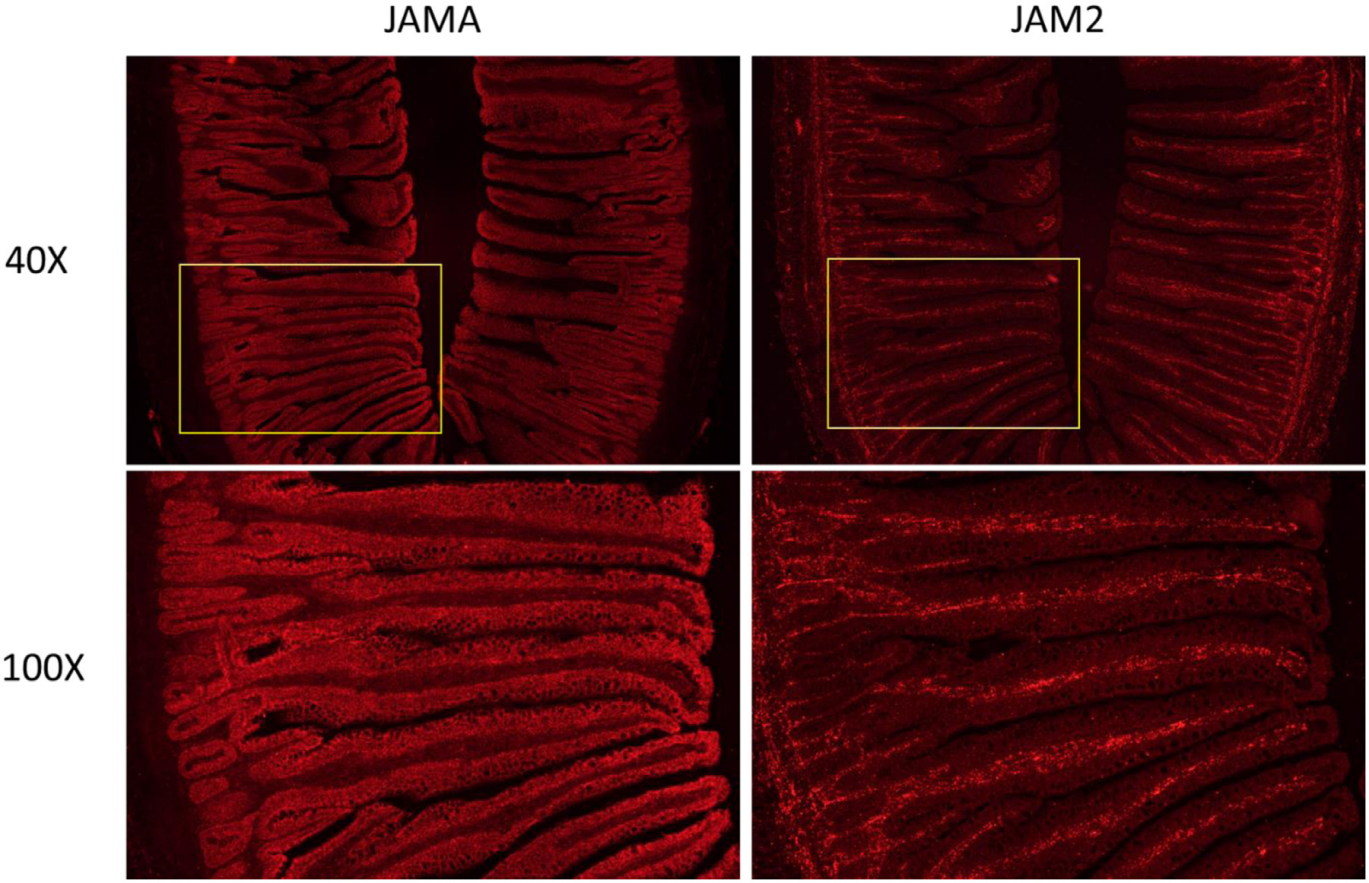
In situ hybridization of JAMA and JAM2 mRNA in jejunal tissue. Serial sections of the jejunum from a broiler at 21 d of age were fixed in formaldehyde and embedded in paraffin. In situ hybridization was performed using custom probes for chicken JAMA and JAM2 and the RNAscope 2.5 HD Assay-RED. Sections were counterstained with hematoxylin. Images were captured at 40× and 100× using fluorescence with a Nikon Eclipse 80i microscope and a Nikon DS-Ri2 camera. The yellow rectangle in the 40× image is the approximate region shown in the 100× image. Abbreviations: JAM2, junctional adhesion molecule 2; JAMA, junctional adhesin molecule A.

The ISH staining patterns for JAMA and JAM2 mRNA in the jejunum from a 21 d old broiler are shown in Figure 1. JAMA and JAM2 mRNA show complementary staining patterns. JAMA mRNA was strongly expressed in the epithelial cells of the villi and crypts. By contrast JAM2 mRNA was present in cells in the center of the villi. These cells form the vascular system of the villi, which transport biomolecules to and from the cells lining the villi. JAM2 mRNA was also present in cells in the lamina propria. These results are consistent with those observed in mammals, where JAMA mRNA is expressed in epithelial cells in the intestine and JAM2 mRNA is expressed in endothelial cells, such as blood vessels in the brain (Hartmann et al., 2020).

A number of studies have analyzed the effect of environmental conditions, probiotics or biomolecules on mRNA abundance of the TJ proteins in the OCLN, CLDN, ZO, and JAM families as a measure of intestinal barrier function (Barekatain et al., 2019; Goo et al., 2019; Park et al., 2020; Tabler et al., 2020; Bilal et al., 2021; Lin et al., 2022). The epithelial cells lining the villi and crypt would be the cells in contact with the contents of the intestinal lumen and thus the TJ between these cells would be key to maintaining the intestinal barrier. The results of this study clearly demonstrate that for the JAM family, JAMA and not JAM2 is the key JAM mRNA to assess the TJ between epithelial cells forming the intestinal barrier. JAM2 mRNA, however, would examine changes in the cells that line the vasculature, which could be important for assessing the effect of environmental conditions, probiotics or biomolecules on the vascular system.

## References

[bib0001] Awad W.A., Hess C., Hess M. (2017). Enteric pathogens and their toxin-induced disruption of the intestinal barrier through alteration of tight junctions in chickens. Toxins.

[bib0002] Barekatain R., Nattrass G., Tilbrook A.J., Chousalkar K., Gilani S. (2019). Reduced protein diet and amino acid concentration alter intestinal barrier function and performance of broiler chickens with or without synthetic glucocorticoid. Poult. Sci..

[bib0003] Bilal M., Si W., Barbe F., Chevaux E., Sienkiewicz O., Zhao X. (2021). Effects of novel probiotic strains of *Bacillus pumilus* and *Bacillus subtilis* on production, gut health, and immunity of broiler chickens raised under suboptimal conditions. Poult. Sci..

[bib0004] Federation for Animal Science Societies (2020).

[bib0005] Goo D., Kim J.H., Choi H.S., Park G.H., Han G.P., Kil D.Y. (2019). Effect of stocking density and sex on growth performance, meat quality, and intestinal barrier function in broiler chickens. Poult. Sci..

[bib0006] Hartmann C., Schwietzer Y.A., Otani T., Furuse M., Ebnet K. (2020). Physiological functions of junctional adhesion molecules (JAMs) in tight junctions. Biomembranes.

[bib0007] Lin Y., Teng P.Y., Olukosi O.A. (2022). The effects of xylo-oligosaccharides on regulating growth performance, nutrient utilization, gene expression of tight junctions, nutrient transporters, and cecal short chain fatty acids profile in *Eimeria*-challenged broiler chickens. Poult. Sci..

[bib0008] Park I., Lee Y., Goo D., Zimmerman N.P., Smith A.H., Rehberger T., Lillehoj H.S. (2020). The effects of dietary *Bacillus subtilis* supplementation, as an alternative to antibiotics, on growth performance, intestinal immunity, and epithelial barrier integrity in broiler chickens infected with *Eimeria maxima*. Poult. Sci..

[bib0009] Tabler T.W.E.S.Greene, Orlowski S.K., Hiltz J.Z., Anthony N.B., Dridi S. (2020). Intestinal barrier integrity in heat-stressed modern broilers and their ancestor wild jungle fowl. Front. Vet. Sci..

[bib0010] Zihni C., Mills C., Matter K., Balda M.S. (2016). Tight junctions: from simple barriers to multifunctional molecular gates. Nature Rev. Mol. Cell Biol..

